# Supplementary figures and images for: English verb regularization in books and tweets
Source: PLoS One. 2018 Dec 28;13(12):e0209651. doi: 10.1371/journal.pone.0209651 (PMC6310258; doi:10.1371/journal.pone.0209651)

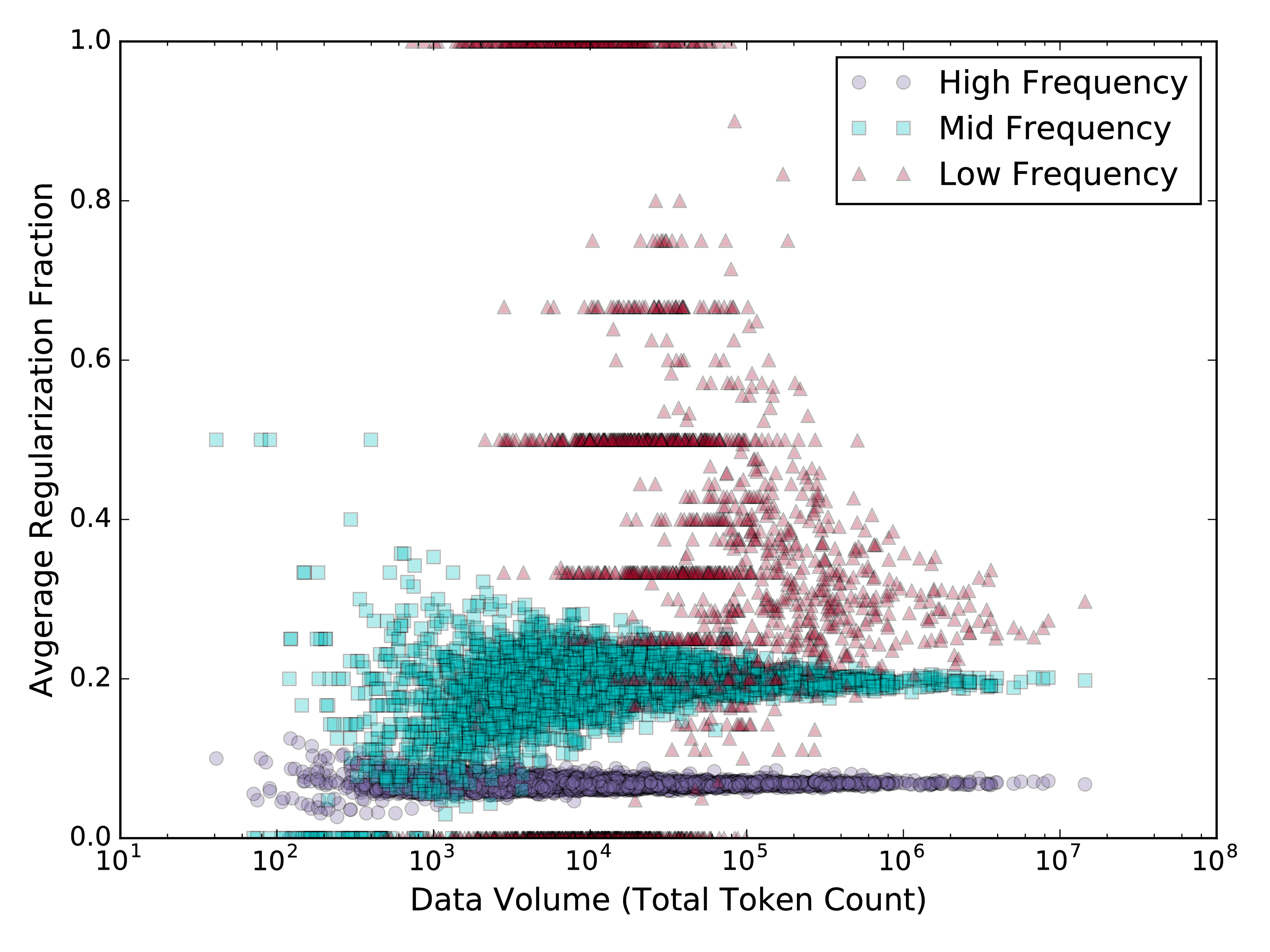

Supplement: S1 Fig — Verbs with a token count in the interval [106, 108] in the Twitter dataset from row (IV) of Table 1 in Sec. 2 are considered ‘high frequency’, those in the interval [104, 106) are ‘mid frequency’, and those in the interval [102, 104) are low frequency. The bins contain 37, 55, and 14 verbs, respectively. For each county (with at least 40 total tokens), the average regularization fraction of the verbs in each of the three bins is calculated (if it is not empty) and plotted against the total token count for all verbs for that county. (TIFF) [file pone.0209651.s001.tiff]

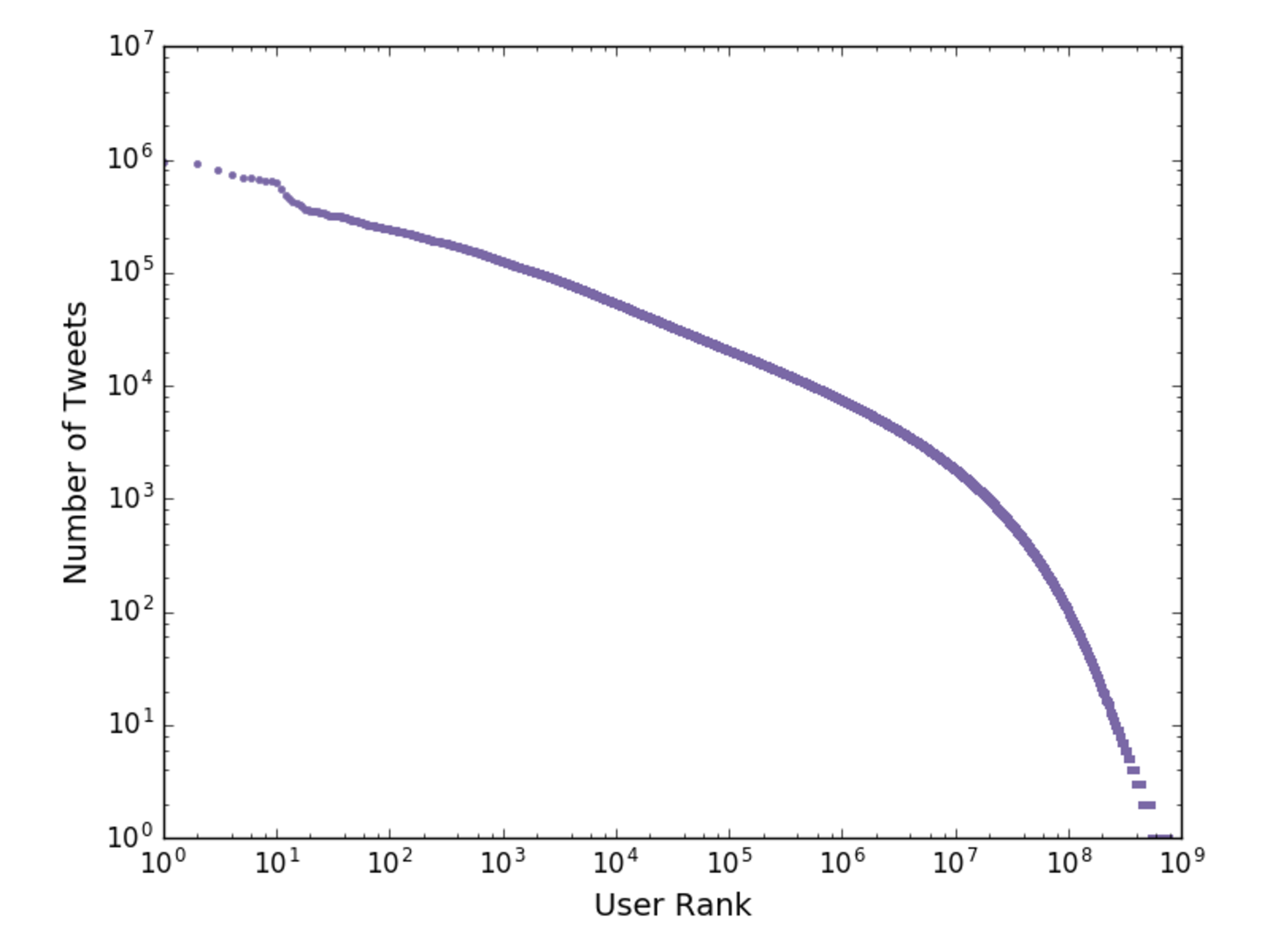

Supplement: S2 Fig — Users are ranked by their total number of tweets along the horizontal axis and the vertical axis gives the total number of tweets we have associated with each user’s account. (TIFF) [file pone.0209651.s002.tiff]
