# Supplementary material for: English verb regularization in books and tweets
Source: PLoS One. 2018 Dec 28;13(12):e0209651. doi: 10.1371/journal.pone.0209651 (PMC6310258; doi:10.1371/journal.pone.0209651)
Supplement: S1 Appendix — (PDF) [file pone.0209651.s003.pdf]

Appendix B: Details on User Location Matching

To study regularization by county, we extracted location information from the user-provided location information, which was entered as free text in the user's biographical profile. To do this, for each tweet we first checked if the location field was populated with text. If so, we then split the text on commas, and checked whether there were two tokens separated by a comma. If so, we made the assumption that it might be of the form 'city, state'. Then we used a python package called uszipcode, which can be found here: pythonhosted.org/uszipcode/. We used the package's method to search by city and state. If the package returned a location match, we used the returned latitude and longitude to determine which county the detected city belonged to.

The package allows for fuzzy matching, meaning the city and state do not have to be spelled correctly, and it allows for the state to be fully spelled out or be an abbreviation. In the source code of the package there was a hard coded confidence level of 70 for the fuzzy matching. We modified the source code so that the confidence level was an input to the method, and running tests found we were satisfied with a confidence level of 91. We checked by hand the matches of 1000 tweets that this method returned a match for, 100 from each year in the dataset, and found the only potential error in these matches was when the user typed in 'Long Island, NY', or a similar variant. For this, the package returned Long Island City, NY, which is on Long Island, but there are multiple counties on Long Island, so the user may actually live in a different county. None of the other 1000 tweets were inappropriately or ambiguously assigned.
