# Supplementary material for: English verb regularization in books and tweets
Source: PLoS One. 2018 Dec 28;13(12):e0209651. doi: 10.1371/journal.pone.0209651 (PMC6310258; doi:10.1371/journal.pone.0209651)
Supplement: S1 Table — The Token Count column gives the sum of all the tokens for the past tense forms of the verb, both regular and irregular, in our Twitter dataset (see row (I) of Table 1 in Sec. 2). (PDF) [file pone.0209651.s004.pdf]

### Appendix A: Table of Verb Forms

| Verb | Regular Preterit & Past Participle | Irregular Preterit | Irregular Past Participle | Token Count |
|---|---|---|---|---|
| abide | abided | abode | abode | 146,566 |
| alight | alighted | alit | alit | 56,306 |
| arise | arised | arose | arisen | 164,134 |
| awake | awaked | awoke | awoken, awoke | 423,359 |
| become | becomed | became | become | 50,664,026 |
| begin | beginned | began | begun | 5,942,572 |
| bend | bended | bent | bent | 4,777,019 |
| beseech | beseeched | besought | besought | 3,390 |
| bleed | bleeded | bled | bled | 252,225 |
| blend | blended | blent | blent | 436,029 |
| bless | blessed | blest | blest | 22,547,387 |
| blow | blowed | blew | blown | 9,155,246 |
| break | breaked | broke | broken | 54,506,810 |
| breed | breeded | bred | bred | 1,040,854 |
| bring | bringed | brought | brought | 15,303,318 |
| build | builded | built | built | 8,521,553 |
| burn | burned | burnt | burnt | 7,457,942 |
| buy | buyed | bought | bought | 24,841,526 |
| catch | catched | caught | caught | 24,891,188 |
| choose | choosed | chose | chosen | 10,290,205 |
| clap | clapped | clapt | clapt | 405,837 |
| climb | climbed | clomb, clom | clomben | 635,122 |

Continued on next page

TABLE A1: A tabulation of all verb forms used in this study. The Token Count column gives the sum of all the tokens for the past tense forms of the verb, both regular and irregular, in our Twitter dataset (see row (I) of Tab. I in Sec. II).

TABLE A1: (continued)

| Verb | Regular<br>Preterit & Past Participle | Irregular<br>Preterit | <br>Past Participle | Token Count |
|---|---|---|---|---|
| cling | clinged | clung | clung | 49,742 |
| creep | creeped | crept | crept | 698,405 |
| deal | dealed | dealt | dealt | 1,181,974 |
| dig | digged | dug | dug | 941,656 |
| dream | dreamed | dreamt | dreamt | 2,794,060 |
| drink | drinked | drank | drunk, drank | 37,295,703 |
| drive | drived | drove | driven | 5,745,497 |
| dwell | dwelled | dwelt | dwelt | 25,725 |
| eat | eated | ate | eaten | 25,084,758 |
| fall | falled | fell | fallen | 25,224,815 |
| fight | fighted | fought | fought | 3,625,297 |
| find | finded | found | found | 80,709,195 |
| flee | fleed | fled | fled | 405,295 |
| freeze | freezed | froze | frozen | 7,454,847 |
| get | getted | got | got, gotten | 500,591,203 |
| give | gived | gave | given | 58,697,198 |
| grow | growed | grew | grown | 17,951,273 |
| hang | hanged | hung | hung | 3,991,956 |
| hear | heared | heard | heard | 52,605,822 |
| hide | hided, hidded | hid | hid, hidden | 7,829,276 |
| hold | holded | held | held | 10,080,725 |
| inlay | inlayed | inlaid | inlaid | 44,811 |
| keep | keeped | kept | kept | 11,785,131 |
| | | | | Continued on next page |

TABLE A1: A tabulation of all verb forms used in this study. The Token Count column gives the sum of all the tokens for the past tense forms of the verb, both regular and irregular, in our Twitter dataset (see row (I) of Tab. I in Sec. II).

TABLE A1: (continued)

| Verb | Regular<br>Preterit & Past Participle | Irregular<br>Preterit | <br>Past Participle | Token Count |
|---|---|---|---|---|
| kneel | kneeled | knelt | knelt | 83,765 |
| know | knowed | knew | known | 58,175,701 |
| lay | layed | laid | laid | 5,828,898 |
| leap | leaped | leapt | leapt | 91,620 |
| learn | learned | learnt | learnt | 18,134,586 |
| lose | losed | lost | lost | 72,695,892 |
| mean | meaned | meant | meant | 26,814,977 |
| pay | payed | paid | paid | 21,150,031 |
| plead | pleaded | pled | pled | 193,553 |
| ride | rided | rode | ridden | 1,710,109 |
| seek | seeked | sought | sought | 888,822 |
| sell | selled | sold | sold | 14,251,612 |
| send | sended | sent | sent | 26,265,441 |
| shake | shaked | shook | shaken | 3,223,316 |
| shoe | shoed | shod | shod | 47,780 |
| shrink | shrinked | shrank, shrunk | shrunk, shrunken | 296,188 |
| sing | singed | sang, sung | sung | 6,767,707 |
| sink | sinked | sank, sunk | sunk, sunken | 927,419 |
| slay | slayed | slew | slain | 2,153,981 |
| sleep | sleeped | slept | slept | 9,252,446 |
| slide | slided | slid | slid | 530,659 |
| sling | slinged | slung | slung | 38,320 |
| slink | slinked | slunk | slunk | 5,772 |

Continued on next page

TABLE A1: A tabulation of all verb forms used in this study. The Token Count column gives the sum of all the tokens for the past tense forms of the verb, both regular and irregular, in our Twitter dataset (see row (I) of Tab. I in Sec. II).

TABLE A1: (continued)

| Verb | Regular | Irregular | | Token Count |
|---|---|---|---|---|
| | Preterit & Past Participle | Preterit | Past Participle | |
| smell | smelled | smelt | smelt | 1,089,814 |
| smite | smitted, smited | smote | smitten, smote | 176,768 |
| sneak | sneaked | snuck | snuck | 797,337 |
| speak | speaked | spoke | spoken | 8,502,050 |
| speed | speeded | sped | sped | 216,062 |
| spell | spelled | spelt | spelt | 3,812,137 |
| spend | spended | spent | spent | 17,603,781 |
| spill | spilled | spilt | spilt | 1,627,331 |
| spin | spinned | spun | spun | 342,022 |
| spoil | spoiled | spoilt | spoilt | 3,891,576 |
| spring | springed | sprang, sprung | sprung | 626,400 |
| stand | standed | stood | stood | 3,942,812 |
| steal | stealed | stole | stolen | 11,884,934 |
| sting | stinged | stung | stung | 391,053 |
| stink | stinked | stank, stunk | stunk | 1,556,197 |
| stride | strided | strode | stridden | 17,811 |
| strike | striked | struck | struck, stricken | 2,167,165 |
| strip | stripped | stript | stript | 837,967 |
| strive | strived | strove | striven | 33,705 |
| swear | sweared | swore | sworn | 1,902,662 |
| sweep | sweeped | swept | swept | 931,245 |
| swim | swimmed | swam | swum | 356,842 |
| swing | swinged | swung | swung | 295,360 |

Continued on next page

TABLE A1: A tabulation of all verb forms used in this study. The Token Count column gives the sum of all the tokens for the past tense forms of the verb, both regular and irregular, in our Twitter dataset (see row (I) of Tab. I in Sec. II).

TABLE A1: (continued)

| Verb | Regular | Irregular | | Token Count |
|---|---|---|---|---|
| | Preterit & Past Participle | Preterit | Past Participle | |
| take | taked | took | taken | 83,457,822 |
| teach | teached | taught | taught | 9,379,039 |
| tear | teared | tore | torn | 4,238,865 |
| tell | telled | told | told | 71,562,969 |
| thrive | thrived | throve | thriven | 43,612 |
| throw | throwed | threw | thrown | 13,197,226 |
| tread | treaded | trod | trodden | 56,624 |
| vex | vexed | vext | vext | 139,411 |
| wake | waked | woke | woken | 30,796,918 |
| wear | weared | wore | worn | 8,552,191 |
| weep | weeped | wept | wept | 200,690 |
| win | winned | won | won | 45,276,202 |
| wind | winded | wound | wound | 1,340,267 |
| wring | wringed | wrung | wrung | 29,141 |
| write | writed | wrote | written, writ, wrote | 23, 926, 025 |
